# Evaluation of the zinc-fingers and homeoboxes 3 as a potential biomarker for prognosis prediction in lung adenocarcinoma

**DOI:** 10.3389/fmed.2025.1728286

**Published:** 2025-12-17

**Authors:** Yanjie You, Wenmei Li, Ling Gao, Tiantian Li, Xiaoli Zhang, Xiaomei Luo

**Affiliations:** 1 Department of Gastroenterology, People’s Hospital of Ningxia Hui Autonomous Region, Ningxia Medical University, Yinchuan, China; 2 Department of Blood Transfusion, People’s Hospital of Ningxia Hui Autonomous Region, Ningxia Medical University, Yinchuan, China

**Keywords:** zinc-fingers and homeoboxes, lung adenocarcinoma, prognosis, data mining, immunohistochemistry

## Abstract

**Background:**

Lung cancer is one of the leading malignant cancers in the world with high incidence and mortality and lung adenocarcinoma (LUAD) is its major histologic subtype. Molecular biomarkers that hold prognostic value and therapeutic implication are required to evaluate disease development and enhance the survival outcomes of cancer patients.

**Materials and methods:**

The current study performed bioinformatics analysis via various online datasets and immunohistochemistry to examine the expression status of the zinc-fingers and homeoboxes (ZHX) family members as well as their prognostic implications in lung cancer as well as in its subtypes.

**Results:**

We observed that high mRNA levels of ZHX factors indicated favorable overall survival in patients with lung cancer. Subgroup analysis demonstrated notable associations between the expression levels of ZHX family members and survival outcomes in select patient cohorts. Immunohistochemistry confirmed that low ZHX3 protein expression had stronger correlation with malignant properties and unfavorable OS in LUAD patients. Multivariate analysis revealed that ZHX3 expression was as an independent predictor for prognosis.

**Conclusion:**

Thus, we supposed that ZHX3 might be a potential survival biomarker in outcome prediction for LUAD.

## Graphical Abstract

**Figure:**
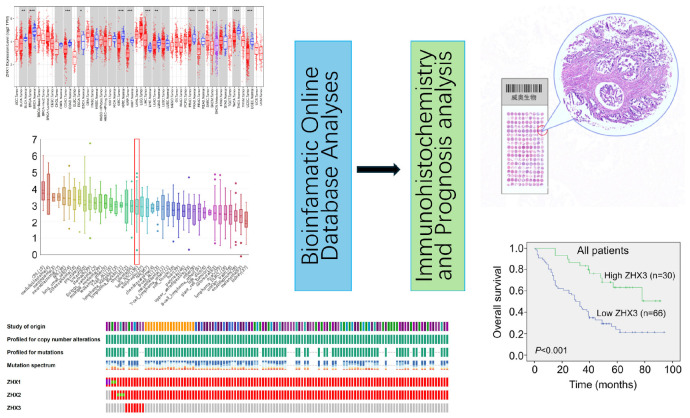
Web-based online bioinformatic analyses and immunohistochemical assessments were conducted to examine the association between transcriptional/protein expression levels of ZHX factors and the corresponding prognostic significance in lung cancer.

## Introduction

Owing to a surprising increase as 15% of all new cancer diagnoses and more than 1.3 million cases diagnosed each year, lung cancer remains the second most frequent cancer worldwide both in incidence and in cancer-related death (1, 2). Despite constant advances in diagnosis and therapeutic technology in recent decades, the survival outcomesh of patients diagnosed with lung cancer remain unsatisfactory (2). Precise molecular mechanisms underlying the development of lung cancer are still elusive. Hence, it is important to develop robust novel biomarkers for patients with lung cancer, to improve survival outcomes.

Through our integrative efforts in discovering new biomarkers whose transcriptional expression significantly correlated with cancer patient survivals by *in silico* bioinformatics approaches, we observed that the zinc-fingers and homeoboxes (ZHX) family members, including ZHX1, ZHX2 and ZHX3, may be in the spotlight (3–5). ZHX family members have been identified as a set of transcription repressors which contain two amino-terminal C2-H2 zinc-finger motifs and four or five carboxy-terminal DNA-binding homeodomains and function through interacting with the A subunit of the nuclear factor Y (NF-YA) and forming homodimers and heterodimers reciprocally (6–11). Multiple studies have reported that ZHX factors are important transcriptional regulators in the events such as proliferation and differentiation of hematopoietic cells and mesenchymal stem cells, as well as maintenance of neural progenitors (6, 12, 13). Dysregulated expression of ZHX family members has also been found to be associated with development of various disorders, e.g., neurological, hematological and glomerular diseases (14–16). Results from the related studies also demonstrated that ZHX factors may associate with the cancer initiation and development (6). The essential functions of ZHX factors provide the possibility for the ZHX factors as potential biomarkers for screening, diagnosis, survival estimation and therapeutic effect assessment. As yet, nevertheless, the prognostic implications of ZHX factors in lung cancer have been poorly examined. In the current study, we utilized bioinformatic analyses to investigate the prognostic values of ZHX factors in lung cancer, through web-based expression and survival analyses. Further, we characterized ZHX3 protein expression profile to confirm its prognostic impacts using a cohort of 96 tumor samples by immunohistochemistry.

## Materials and methods

### Oncomine database analysis

The mRNA levels of ZHX factors in a set of diverse cancers were analyzed using the Oncomine online database,^1^ which provides 715 datasets and 86,733 clinical specimens to conduct relevant research according to genomic expression analyses. When mRNA expression levels of target genes in tumor samples were compared to those in normal tissues, a fold-change ≥ 2 along with *P* < 0.01 was considered as statistically significant (17).

### Tumor IMmune Estimation Resource database analysis

The TIMER online database^2^ has been recognized as an advanced and comprehensive resource to systematically examine immune infiltrates in cancers (18). In the present study, the mRNA expression levels of ZHX factors in multiple cancer types were calculated through the TIMER database analysis.

### Cancer Cell Line Encyclopedia database analysis

The mRNA expression levels of ZHX factors in different types of cancer cell lines were assessed using the CCLE online database,^3^ which is an online data collection of gene expression, copy numbers and large-scale genetic sequences from 1,457 human cancer cell lines (19).

### Kaplan-Meier Plotter survival analysis

The impacts of ZHX mRNA expression levels on patient outcomes were examined using the Kaplan-Meier Plotter online database,^4^ which contained the gene expression information and survival data from 1927 patients with lung cancer (20, 21). To evaluate the overall survival (OS) and post-progression survival (PPS), clinical specimens were separated into high and low expression groups by auto-selected best cut-off for gene expression analysis. After the target gene probe was participated in the database, Kaplan-Meier plots were achieved by survival analyses. The hazard ratio (HR) with 95% confidence intervals (CI) and the log-rank *P*-values were analyzed and displayed on the webpage.

### cBioPortal cancer genomics database analysis

The impacts of genomic alterations of ZHX factors including gene copy-number variance, deletion and mutations on OS and disease-free survival (DFS) of cancer patients were detected using the cBioPortal online database^5^ (22, 23).

### Immunohistochemistry and evaluation

One tissue microarray chip (HLugA180Su07) was purchased from Outdo Biotech Co., Ltd. (Shanghai, China), including 98 primary lung adenocarcinoma (LUAD) tissues along with 82 paired adjacent non-cancerous tissues from patients undergoing curative surgery between February 2008 and June 2010. All samples were surgical cases, and histopathologically and clinically confirmed. Tumor grade and stage were classified in accordance with the Union of International Cancer Control (UICC)/American Joint Committee on Cancer (AJCC) pathologic tumor-node-metastasis (TNM) classification, 7th edition (2010). No patients in the current study had experienced preoperative chemotherapy or radiotherapy. Immunohistochemical analysis was performed as previously described (3–5, 24–26). The specimen sections were fixed, deparaffinized, rehydrated, and incubated with a rabbit polyclonal anti-ZHX3 antibody (catalog no. ab84677; dilution, 1:500; Abcam, Cambridge, MA, United States) and an EnVision antibody complex (anti-mouse/rabbit) from an Envision™ Detection kit (OriGene Technologies, Inc., Wuxi, China). Final staining was conducted by incubation with 3,3’-diaminobenzidine (DAB) as the substrate. Nuclei were counterstained with hematoxylin. Ten random 400 × microscopic fields per slide of each specimen were examined by two independent pathologists who were blinded to the clinical information. Staining results were determined using a semi-quantitative method by integrating the percentage and the intensity of positively-stained cells. The mean percentage was classified as follows: 0–25% (0); 26–50% (1); 51–75% (2); and 76–100% (3). The staining intensity was scored as follows: Absent (0); weak (1); moderate (2); and strong (3). The multiplication of these above parameters was employed as the final staining score. The tumor specimens with a final score of < 2 were defined as low ZHX3 expression and those with a score ≥ 2 as high ZHX3 expression.

### Statistical analysis

Statistical analyses were performed using the SPSS 17.0 statistical software package (SPSS Inc., Chicago, IL, United States). The differences in the expression levels of ZHXs were correlated with different clinical variables through Fisher’s exact test or Pearson χ^2^-test, whichever was appropriate. The Kaplan-Meier method with a log-rank test was utilized to examine clinical outcome after surgery. Univariate and multivariate analyses were determined using a Cox proportional hazards regression model to investigate the independent factors that affected patient survival. *P* < 0.05 was considered to indicate a statistically significant difference.

## Results

### Transcriptional levels of ZHX factors in cancers

**FIGURE 1:**
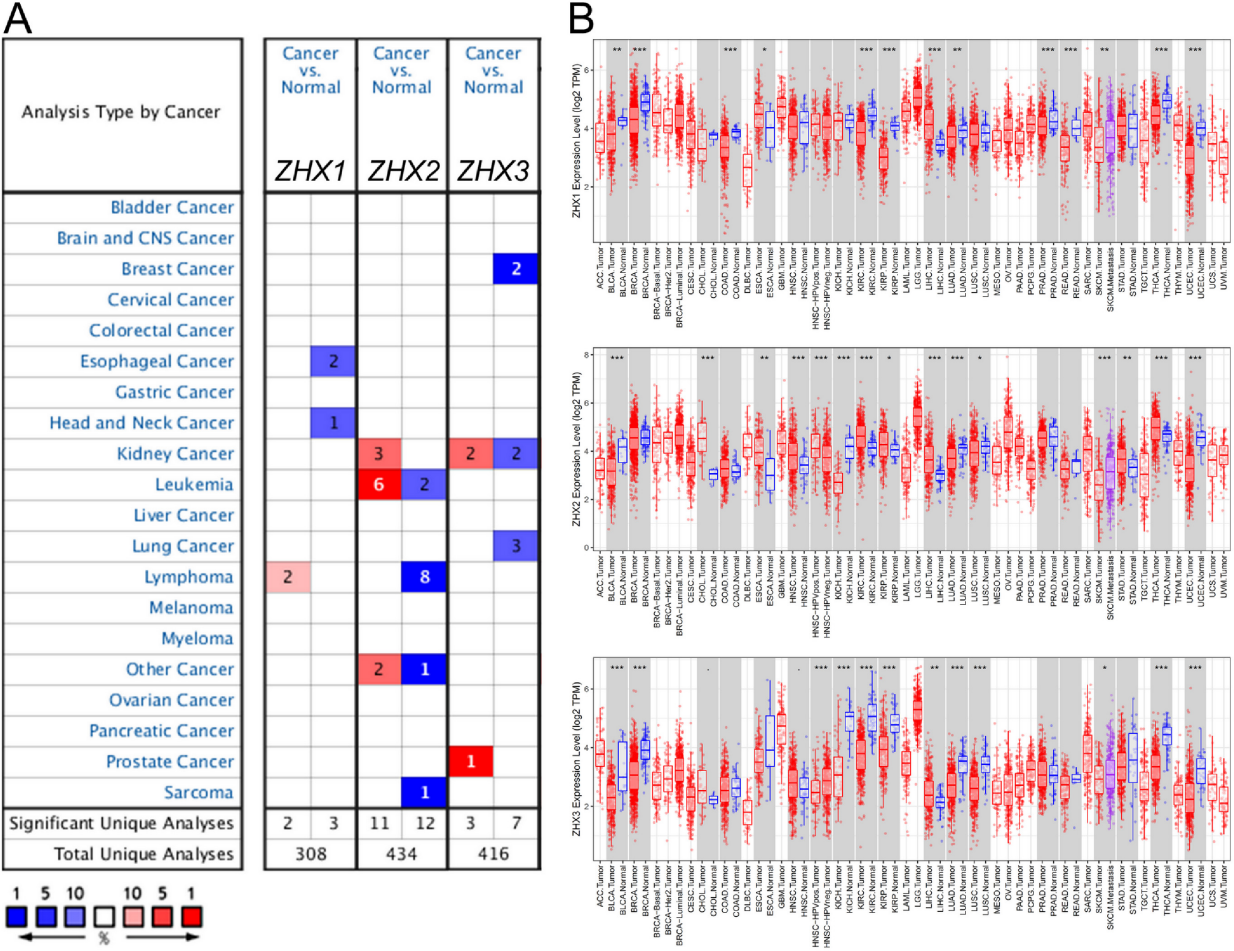
The mRNA expression levels of ZHX factors in diverse types of cancer. **(A)** A graphic produced by the Oncomine online database shows the numbers of datasets with significant overexpression (red) or downregulation (blue) of ZHX factors at the mRNA levels in tumor tissues compared with corresponding normal tissues. The cell color was defined by the best gene rank which was assessed by percentile of target genes in the top of all genes analyzed in each study. **(B)** Transcriptional expression levels of ZHX factors among various types of cancer via TIMER database analysis. *P < 0.05; **P < 0.01, ***P < 0.001. ZHX, zinc-fingers and homeoboxes; TIMER, Tumor IMmune Estimation Resource.

**FIGURE 2:**
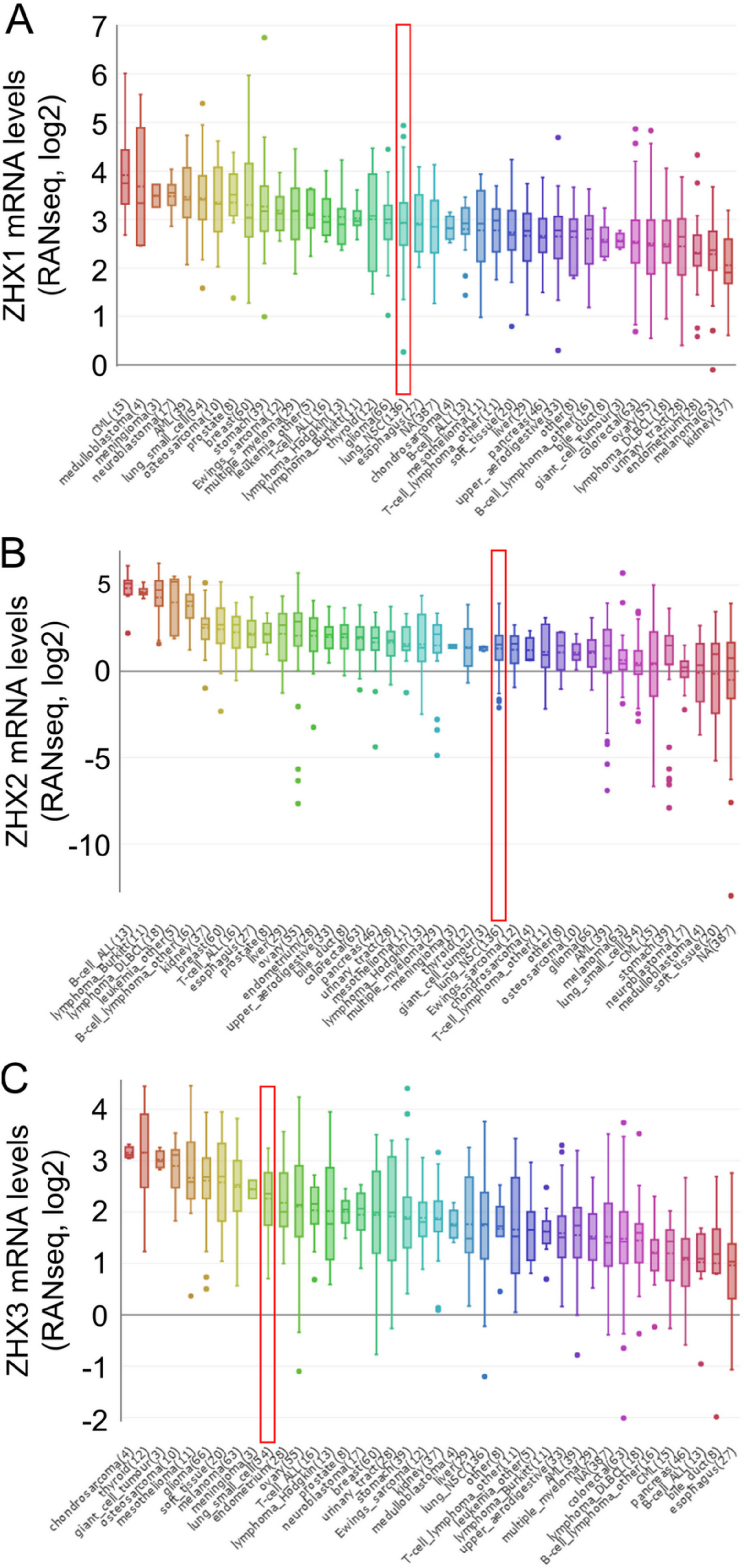
The mRNA expression levels of ZHX factors are distinctive in lung cancer cell lines according to the CCLE database analysis. The mRNA expression levels of **(A)** ZHX1, **(B)** ZHX2 and **(C)** ZHX3 in lung cancer cells ranked the 19th, 25th and 10th highest across diverse types of cancer (shown in red frame). ZHX, zinc-fingers and homeoboxes.

We set out to identify the differences in mRNA expression levels of three ZHX factors between cancer tissues and normal tissues in diverse types of cancer by means of bioinformatics analysis. By using the Oncomine database, we found 308, 434 and 416 unique analyses for ZHX1, ZHX2 and ZHX3, separately (Figure 1A). Three analyses revealed attenuated ZHX1 mRNA expression in cancer tissues than in normal tissues, whereas two other analyses showed an opposite result. ZHX2 mRNA expression was observed to be decreased in 12 analyses but increased in 11 analyses. ZHX3 mRNA expression was found to be downregulated in seven analyses but upregulated in three analyses. Regarding lung cancer, however, only three analyses reported lower ZHX3 expression. We further investigated ZHX3 mRNA expression among various types of cancer using the TIMER online database. Except the comparison of ZHX1 on lung squamous cell carcinoma (LUSC), the mRNA levels of all three ZHX factors was significantly lower in both lung adenocarcinoma (LUAD) and LUSC tissues than in normal tissues (Figure 1B). Additionally, analysis from the CCLE database demonstrated that the mRNA levels of ZHX1, ZHX2 and ZHX3 in lung cancer cell listed in the 19th, 25th, and 10th highest positions across all types of cancer, respectively (Figure 2).

### Association between mRNA expression levels of ZHX factors and prognosis

**FIGURE 3:**
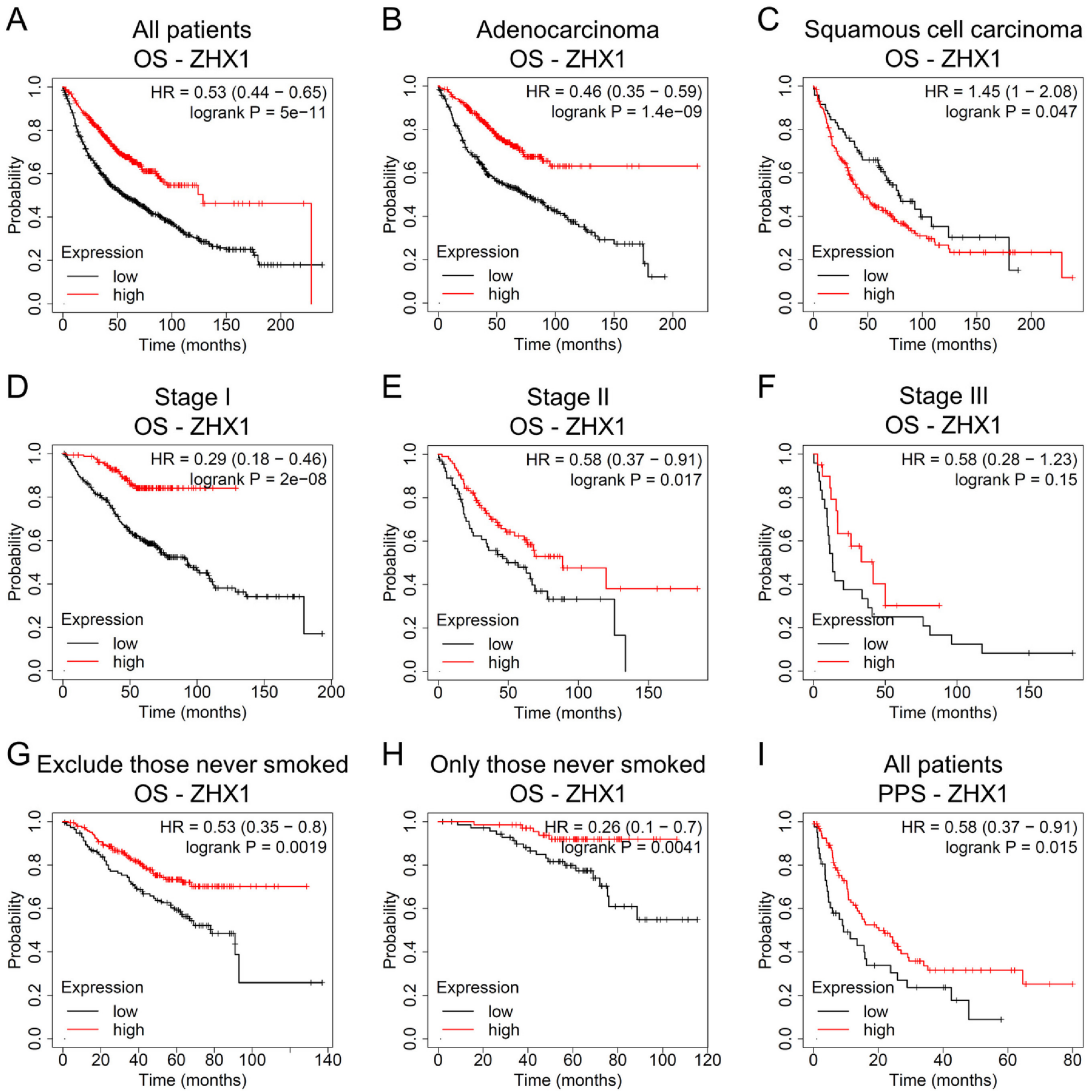
Association between ZHX1 mRNA levels and prognosis in lung cancer via Kaplan-Meier Plotter survival analysis. **(A)** OS analysis of ZHX1 in all patients. OS analysis of ZHX1 in patients with **(B)** adenocarcinoma and **(C)** squamous cell carcinoma. OS analysis of ZHX1 in patients with **(D)** Stage I, **(E)** Stage II and **(F)** Stage III tumors. OS analysis of ZHX1 in patients **(G)** excluding those never smoked and **(H)** only those never smoked. **(I)** PPS analysis of ZHX1 in all patients. ZHX, zinc-fingers and homeoboxes; OS, overall survival; PPS, post-progression survival.

We next determined whether ZHX factors may of value in predicting survival outcomes in patients with lung cancer via Kaplan-Meier Plotter survival analysis. Increased ZHX1 mRNA expression was found to be associated with a better OS rate in patients with lung cancer (Figure 3A). Subgroup analyses revealed that high mRNA expression of ZHX1 predicted a longer OS in patients with LUAD (Figure 3B), but exhibited a shorter OS in patients with LUSC (Figure 3C). High ZHX1 mRNA level indicated a favorable OS in patients with Stage I tumors and Stage II tumors (Figures 3D, E), but not in those with Stage III tumors (Figure 3F). Increased ZHX1 was also associated with longer OS times in patients excluding those never smoked and in patients only those never smoked (Figures 3G, H). Of note, high ZHX1 levels displayed an improved PPS rate in patients with lung cancer (Figure 3I).

**FIGURE 4:**
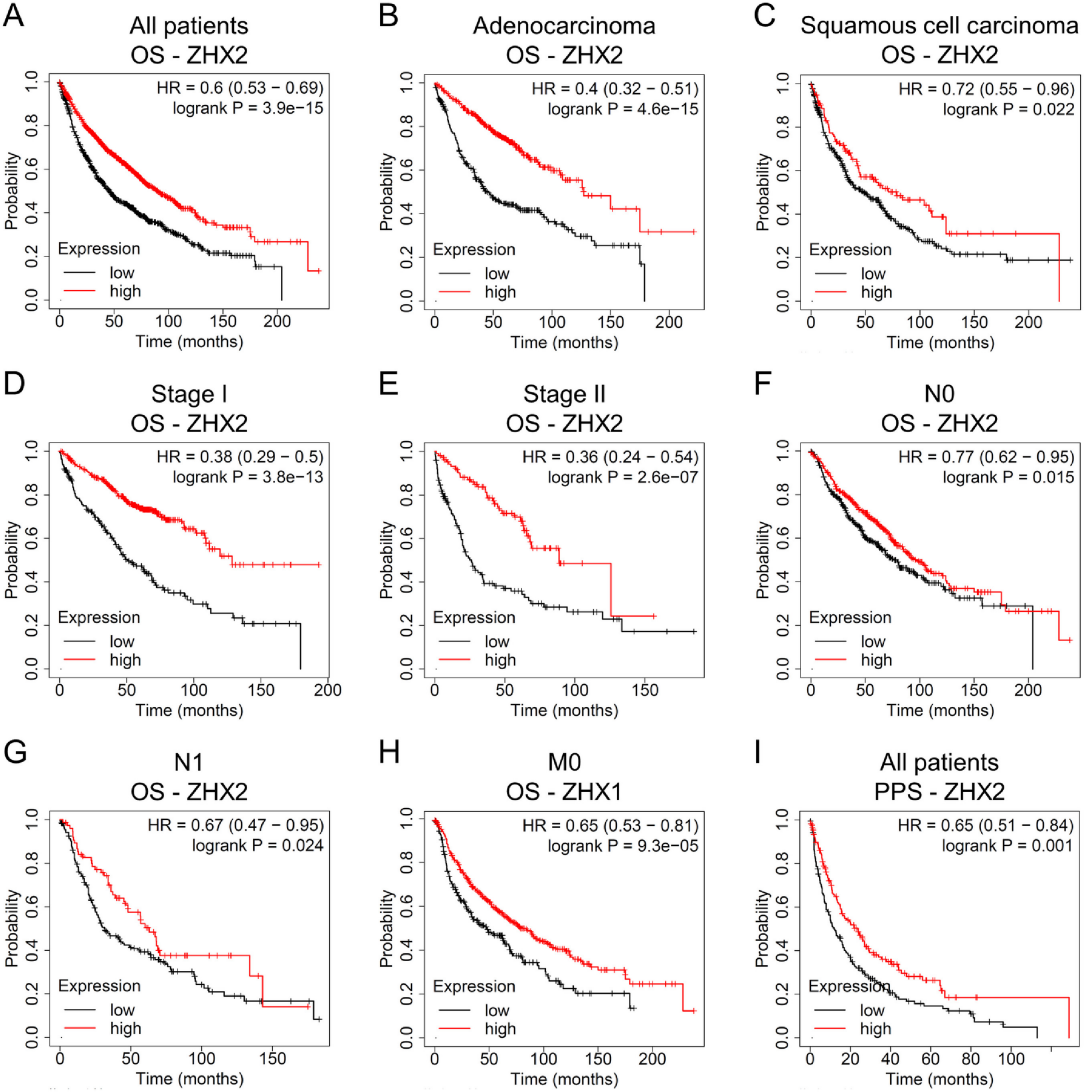
Association between ZHX2 mRNA levels and prognosis in lung cancer via Kaplan-Meier Plotter survival analysis. **(A)** OS analysis of ZHX2 in all patients. OS analysis of ZHX2 in patients with **(B)** adenocarcinoma and **(C)** squamous cell carcinoma. OS analysis of ZHX2 in patients with **(D)** Stage I and **(E)** Stage II tumors. OS analysis of ZHX2 in patients with **(F)** N0, **(G)** N1, and **(H)** M0 tumors. **(I)** PPS analysis of ZHX2 in all patients. ZHX, zinc-fingers and homeoboxes; OS, overall survival; PPS, post-progression survival.

High ZHX2 mRNA level was observed to be associated with a better OS rate in patients with lung cancer (Figure 4A). Subgroup analyses suggested that upregulated ZHX2 indicated a favorable OS in patients with LUAD and LUSC (Figures 4B,C). Elevated ZHX2 expression implied a favorable OS in patients with Stage I and Stage II tumors (Figures 4D,E). High ZHX2 expression implied an improved OS in patients with low frequency of lymph node metastases (N0 and N1) (Figures 4F,G). Increased ZHX2 levels also implied a longer OS in patients without distant metastasis (M0) (Figure 4H). Furthermore, elevated ZHX2 exhibited an improved PPS rate in patients with lung cancer (Figure 4I).

**FIGURE 5:**
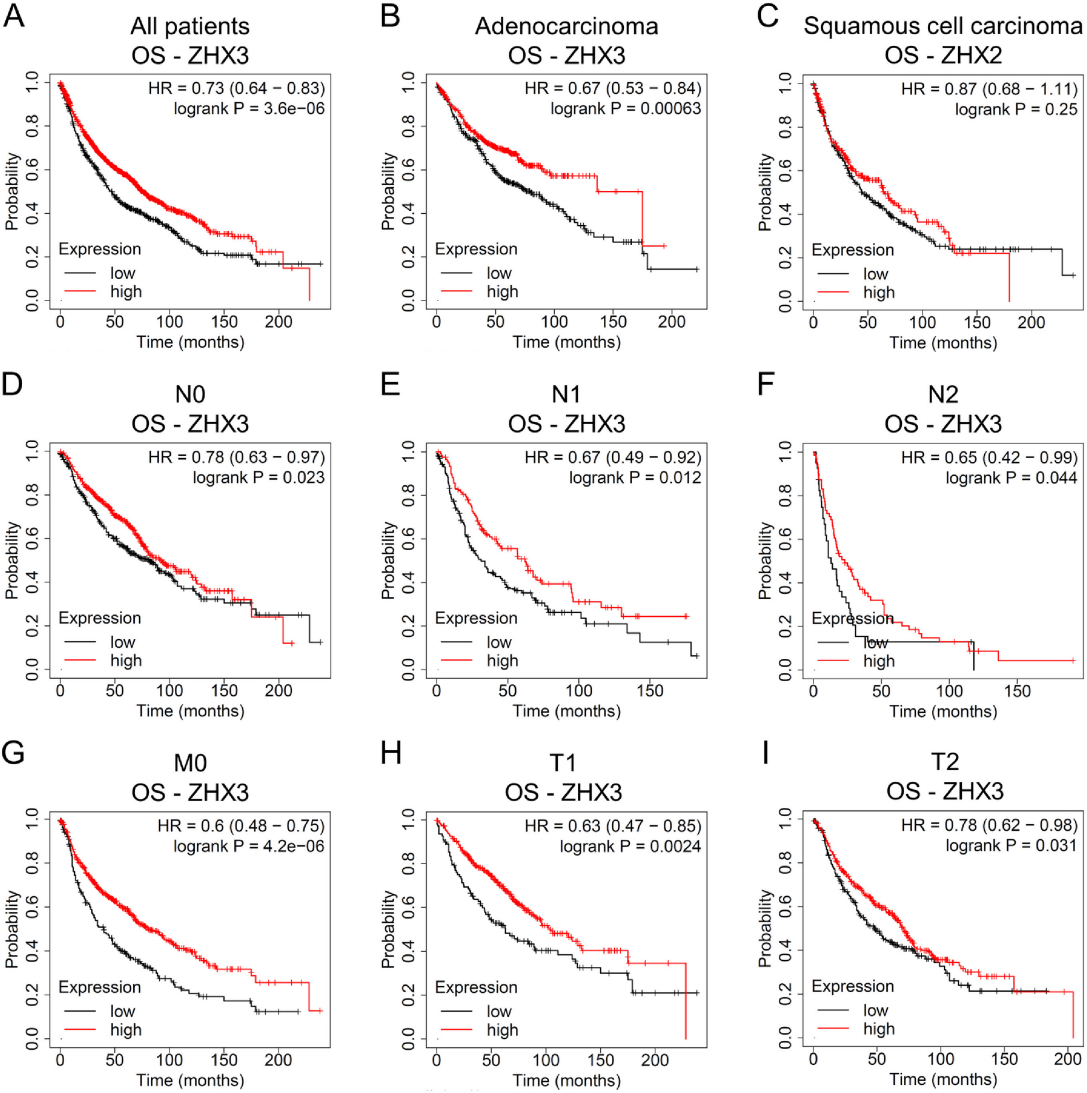
Association between ZHX3 mRNA levels and prognosis in lung cancer via Kaplan-Meier Plotter survival analysis. **(A)** OS analysis of ZHX3 in all patients. OS analysis of ZHX3 in patients with **(B)** adenocarcinoma and **(C)** squamous cell carcinoma. OS analysis of ZHX3 in patients with **(D)** N0, **(E)** N1, **(F)** N2 and **(G)** M0 tumors. OS analysis of ZHX3 in patients with **(H)** T1 and **(I)** T2 tumors. ZHX, zinc-fingers and homeoboxes; OS, overall survival; PPS, post-progression survival.

Increased ZHX3 mRNA expression was shown to be associated with a prolonged OS rate in patients with lung cancer (Figure 5A). Subgroup analyses illustrated that increased ZHX3 level represented longer OS times in patients with LUAD (Figure 5B), but not in those with LUSC (Figure 5C). Increased ZHX3 expression indicated a better OS in patients with and without lymph node metastases (N0, N1, and N2) (Figures 5D–F). High ZHX3 expression also represented a favorable OS in patients without distant metastasis (M0) (Figure 5G). In addition, elevated ZHX3 was associated with a better OS rate in patients with T1 and T2 tumors (Figures 5H,I).

### Association between genomic alterations of ZHX factors and patient survival

**FIGURE 6:**
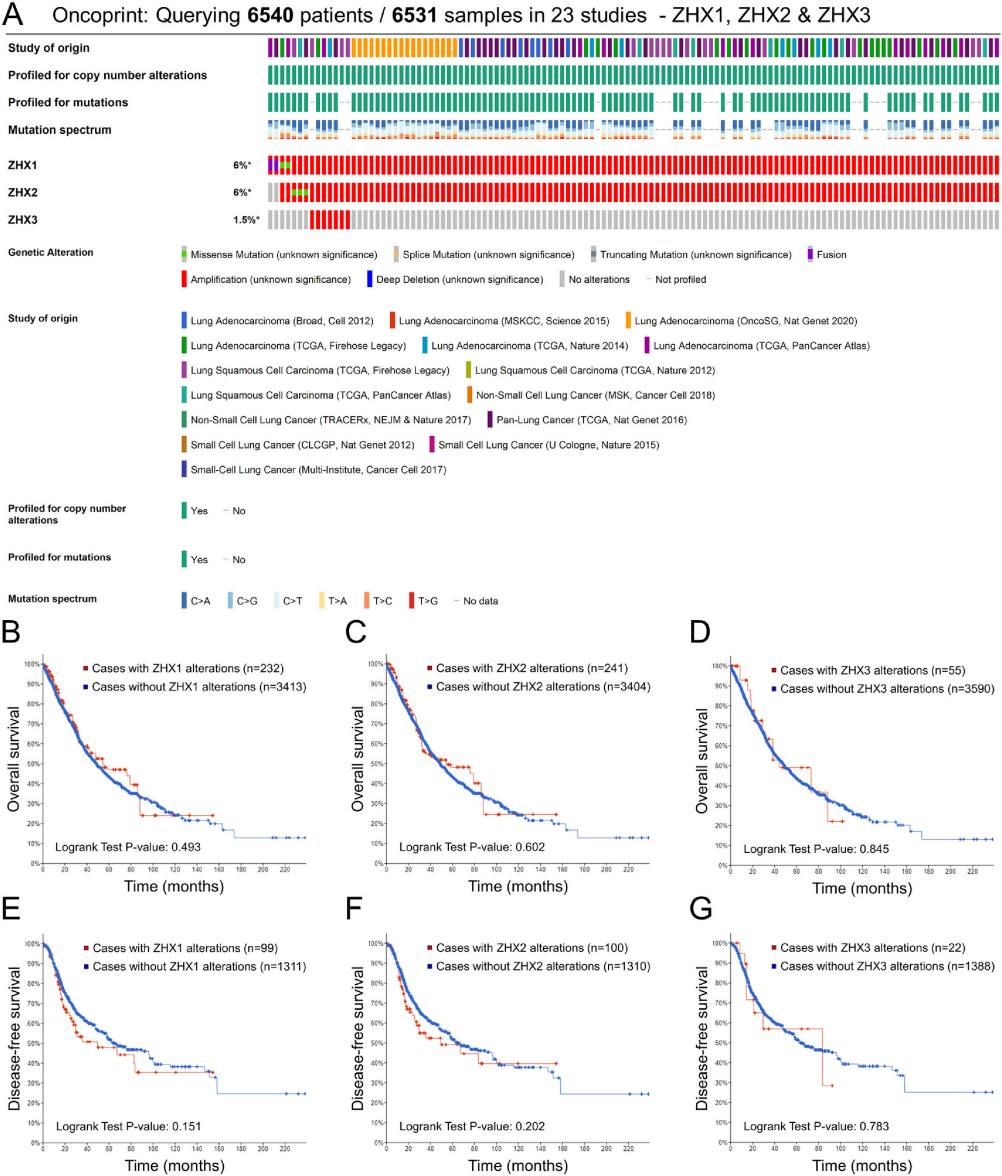
Genomic alterations of ZHX factors and their corresponding associations with patient survival in lung cancer via cBioPortal database analysis. **(A)** The proportion and distribution of samples with genomic alterations in ZHX factors by OncoPrint in cBioPortal. The impacts of genomic alterations of **(B)** ZHX1, **(C)** ZHX2 and **(D)** ZHX3 on OS in patients with lung cancer. The impacts of genomic alterations of **(E)** ZHX1, **(F)** ZHX2 and **(G)** ZHX3 on DFS in patients with lung cancer. ZHX, zinc-fingers and homeoboxes; OS, overall survival; DFS, disease-free survival.

We then characterized the prognostic correlation between genetic alterations of ZHX factors and survival outcomes in patients with lung cancer using the cBioPortal database. The genomic alteration rates for ZHX1, ZHX2, and ZHX3 were 6, 6, and 1.5%, respectively (Figure 6A). Nevertheless, no significant association was found between genomic alterations of ZHX family members and patient survival, as regarding either OS or DFS rates (Figures 6B–G).

### ZHX3 expression is an independent prognostic indicator in LUAD

**FIGURE 7:**
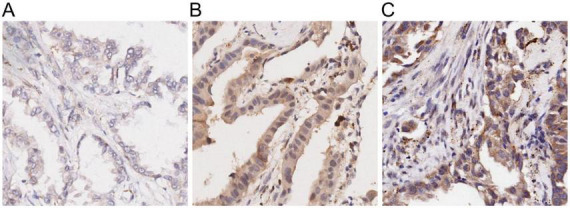
Representative staining of immunohistochemistry for ZHX3 in LUAD tissues. **(A)** Weak staining; **(B)** Moderate staining; and **(C)** Strong staining. Original magnification, 200×. ZHX, zinc-fingers and homeoboxes. LUAD, lung adenocarcinoma.

**FIGURE 8:**
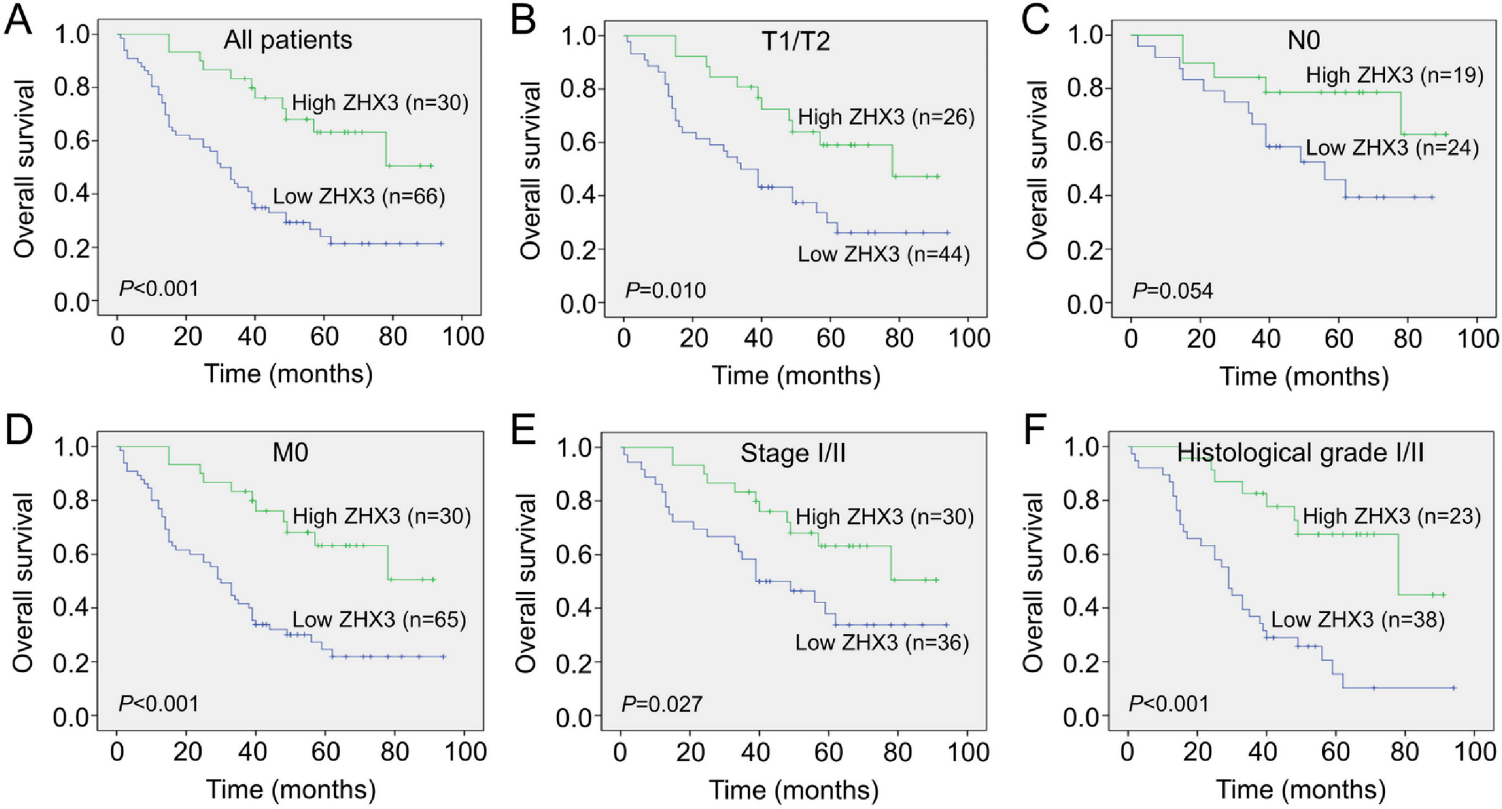
Kaplan-Meier curves comparing the survival outcomes in LUAD patients with high and low ZHX3 protein expression. **(A)** High ZHX3 expression was significantly correlated with a better OS in patients with lung cancer. **(B)** OS curves of ZHX3 expression in patients with T1/T2 tumors. **(C)** OS curves of ZHX3 expression in patients with N0 tumors. **(D)** OS curves of ZHX3 expression in patients with M0 tumors. **(E)** OS curves of ZHX3 expression in patients with Stage I/II tumors. **(F)** OS curves of ZHX3 expression in patients with Histological grade I/II tumors. ZHX, zinc-fingers and homeoboxes; LUAD, lung adenocarcinoma; OS, overall survival.

To support of the above observations, we further investigated the protein expression status of ZHX3 using immunohistochemistry and one tissue microarray chip including 98 LUAD tissues and 82 adjacent non-cancerous tissues, in which two cancer samples were detached in the process of IHC staining. A high level of ZHX3 protein expression was observed in the cytoplasm of tumor cells in 31.3% (30/96) of the LUAD samples tested (Figures 7A–C). Of note is that high ZHX3 protein expression was also found in 56.1% (46/82) of adjacent non-cancerous tissues, which was significantly higher than that in cancer tissues (Table 1). Low ZHX3 expression was observed to be associated with T stage, N stage and clinical stage (Table 2). Kaplan-Meier survival curves showed that patients with high ZHX3 expression exhibited a better OS than those with low ZHX3 expression (Figure 8A). Subgroup analyses demonstrated that high ZHX3 expression predicted an improved OS in patients with T1/T2 tumors (Figure 8B). High ZHX3 expression also represented longer OS times in patients without lymph node metastases (N0) (Figure 8C) and in patients without distant metastasis (M0) (Figure 8D). Moreover, elevated ZHX3 expression suggested a prolonged OS in patients with Stage I/II tumors (Figure 8E) and in patients with Histological grade I/II tumors (Figure 8F). On univariate analyses, patient age, T stage, clinical stage and ZHX3 expression were proven to be responsible for an unfavorable OS (Table 3). Following adjusting the prognostic variables established in univariate analyses, patient age, clinical stage and ZHX3 expression had the independent significance for OS in multivariate analyses (Table 3).

**TABLE 1:** Differential expression of ZHX3 protein in LUAD tissues and adjacent non-cancerous tissues.

Variables	Total	ZHX3 protein expression		Chi-square value	*P*-value
		Low, *n* (%)	High, *n* (%)		
LUAD tissues	96	66 (68.7)	30 (31.3)	11.160	0.001
Non-cancerous tissues	82	36 (43.9)	46 (56.1)		

**TABLE 2:** Relationship between ZHX3 expression and clinicopathological characteristics in lung cancer.

Parameters	No. of patients	ZHX3 protein expression		*P*-value
		Low, *n* (%)	High, *n* (%)	
**Age**				
≤60 years	50	35 (70.0)	15 (30.0)	0.783
>60 years	46	31 (47.5)	15 (52.5)	
**Sex**				
Male	54	41 (75.9)	13 (24.1)	0.085
Female	42	25 (59.5)	17 (40.5)	
**Histological grade**				
I/II	61	38 (62.3)	23 (37.7)	0.072
III	35	28 (80.0)	7 (20.0)	
**Clinical stage (pTNM)**				
I/II	66	36 (54.5)	30 (45.5)	< 0.001
III/IV	30	30 (100.0)	0 (0.00)	
**T stage**				
T1/T2	70	44 (62.3)	26 (37.1)	0.024
T3/T4	24	21 (87.5)	3 (12.5)	
NA	2			
**N stage**				
N0	43	24 (55.8)	19 (44.2)	< 0.001
N1-N3	37	35 (94.6)	2 (5.4)	
NA	16			
**M stage**				
M0	95	65 (68.4)	30 (31.6)	1.000
M1	1	1 (100.0)	0 (0.0)	
**PD-L1**				
Negative	70	50 (71.4)	20 (28.6)	0.518
Positive	13	8 (61.5)	5 (38.5)	
NA	13			
**ALK**				
Negative	65	48 (73.8)	17 (26.2)	0.131
Positive	15	8 (53.3)	7 (46.7)	
NA	16			
**EGFR**				
Negative	62	42 (67.7)	20 (32.3)	0.743
Positive	13	10 (76.9)	3 (23.1)	
NA	21			

NA, not available; PD-L1, programmed death-ligand 1; ALK, anaplastic lymphoma kinase; EGFR, epidermic growth factor receptor.

**TABLE 3:** Univariate and multivariate analyses of the factors correlated with overall survival in patients with lung cancer.

Variables	Univariate analysis		Multivariate analysis	
	HR (95% CI)	*P*-value	HR (95% CI)	*P*-value
Age	0.578 (0.344–0.972)	0.039	0.547 (0.325–0.923)	0.024
T stage	2.141 (1.220–3.754)	0.002	0.997 (0.479–2.076)	0.994
Clinical stage	3.685 (2.145–6.331)	< 0.001	2.570 (1.218–5.422)	0.013
ZHX3 expression	0.307 (0.159–0.593)	< 0.001	0.421 (0.203–0.872)	0.020

HR, hazard ratio; CI, confidence interval.

## Discussion

Despite new discoveries and treatment including targeted agents, e.g., epidermal growth factor receptor inhibitors, dedicated to precision medicine, the overall survival rates of lung cancer patients have not shown significant improvement (27). Patients with localized lung cancer are treated and managed with promise to cure. Nevertheless, the optimal surveillance of these patients for recurrence and metastasis after curative therapy is urgently needed. The utilization of early detection and treatment might result in improved clinical outcomes by image-based surveillance strategies as well as biomarker evaluation (28). The present study is part of our continuous effort to reveal molecular biomarkers with reliability for predicting outcomes for cancer patients. Confirmation of this issue might be critical to direct clinical management of cancers in the future. Thus, the present study using data from public resources as well as immunohistochemistry provide an in-depth insight into the prognostic implication of ZHX family members in patient with lung cancer. Our findings suggest that the expression of ZHX factors may serve as promising prognostic indicators in patients with lung cancer.

ZHX1 has been identified as the first member of the ZHX family, and has been reported as a tumor suppressor in several types of cancer (6–8, 29–33). Nevertheless, recent reports have implied that ZHX1 might serve as an oncogene and its overexpression was correlated with a worse prognosis in cancers (34–36). Thus, the prognostic impact of ZHX1 on various cancer types, even in their subtypes, appears to be contradictory. We recently showed that high ZHX1 expression predicts an unfavorable OS rate in breast cancer but presents favorable OS for gastric cancer, confirming its different roles in different cancer types (3, 4). In the present study, a significant association was observed between ZHX1 mRNA expression and survival outcomes in patients with lung cancer in this study. However, high ZHX1 expression predicted a better OS in patients with LUAD but presented a worse OS in patients with LUSC. We inferred that discrepant cancer specimen sources, histological phenotypes and intrinsic differences in different cancer subtypes may be reasonable to explain this contradictory finding (37). Moreover, a prognostic value of ZHX1 expression was identified in the subgroup analyses, i.e., significant associations between high ZHX1 mRNA levels and longer OS rates in patients with Stage I and II tumors, suggesting that ZHX1 may exert its function as a valuable prognostic predictor only for patients with early-stage disease. Of note is that, in order to provide the more important and significant observations, we preferred to exhibit the selected results with statistically significant difference in the present study.

Similarly, we found that high ZHX2 expression was not only correlated with a prolonged OS, but also indicated a better PPS rate in patient with lung cancer. This founding demonstrates a tumor-suppressor role of ZHX2, consistent with another report in lung cancer suggesting ZHX2 represses proliferation and increases apoptosis of tumor cells via inactivation of p38MAPK signaling (38). Of note, our current finding revealed that increased ZHX2 expression was related with a better OS in patients with Stage I/II, N0/N1, and M0 tumors, suggesting ZHX2 as a valuable prognostic indicator for patients with early-stage disease and low frequency of metastasis. More intensively, it has been reported that ZHX2 suppresses metastasis of thyroid cancer through transcriptional suppression of the S100 calcium binding protein A14 (39). Although these above observations suggest that ZHX2 might serve as a tumor suppressor in lung cancer, ZHX2 expression has been observed to be upregulated in clear cell renal cell carcinoma and facilitate tumorigenesis of in a hypoxia inducible factor-α -independent manner (40, 41). These observations seem consistent with the relevant findings of ZHX2 as an oncogene in gastric cancer, including our previous report (4, 42). In addition, it has been found that ZHX2 may promote HIF1α oncogenic signaling in triple-negative breast cancer (TNBC) and hypoxia-induced phase separation of ZHX2 may alter chromatin looping to promote metastasis (43, 44). ZHX2 has also been found to modulate E-cadherin expression and enrich MET cells to suppress lung metastasis of TNBC (45). All these above data suggest that ZHX2 may have different functions according the specific types of cancer.

Consistent with our recent reports in liver cancer and breast cancer (3, 5), increased ZHX3 expression has been found to be correlated with a favorable OS in patients with lung cancer and may be of value to predict the outcomes of patients with early-stage disease. This result is contrary to the oncogene role of ZHX3 in gastric cancer and bladder urothelial carcinoma (4, 46). To support the findings by bioinformatics analysis, we performed immunohistochemistry to examine ZHX3 protein expression level in LUAD samples and further investigate the corresponding prognostic implication. We observed that low ZHX3 expression were associated with malignant properties and may be an independent factor for prognostic prediction of LUAD. Thus, we conclude that ZHX3 may act as an important role in the progression of LUAD.

There are several limitations to be addressed as regarding the current study. Because the current study performed integrative bioinformatics analyses using a set of online databases, certain search parameters were not available. The limited sample size and restricted follow-up period in the immunohistochemical analysis demands enlarged sample cohort with integral information in our future work. The biological functions and underlying molecular mechanisms of ZHX factors in lung cancer require further validation under well-controlled conditions in cell and/or animal models. Moreover, considering there are some widely-used biomarkers for lung cancer such as EGFR, KRAS, and PD-L1, combination of these factors with ZHX factors may provide more accurate and precise methods for prediction of patient survival.

In the present study, we systematically investigated the expression profile and prognostic implication of ZHX family members in lung cancer. Our results suggest that dysregulation of ZHX factors is involved in disease progression of lung cancer and ZHX3 expression may serve as a novel and promising biomarker for survival prediction in LUAD patients.

## Data Availability

The data presented in the study are deposited in the repository of People’s Hospital of Ningxia Hui Autonomous Region, accession number (2023BEG02001).

## Footnotes

1
http://www.oncomine.org

2
https://cistrome.shinyapps.io/timer

3
http://portals.broadinstitute.org/ccle

4
http://kmplot.com/analysis

5
http://www.cbioportal.org
